# A memristive spiking neuron with firing rate coding

**DOI:** 10.3389/fnins.2015.00376

**Published:** 2015-10-20

**Authors:** Marina Ignatov, Martin Ziegler, Mirko Hansen, Adrian Petraru, Hermann Kohlstedt

**Affiliations:** Nanoelektronik, Technische Fakultät, Christian-Albrechts-Universität zu KielKiel, Germany

**Keywords:** memristive devices, negative differential resistor, spiking neuron, neural coding, neuromorphic systems

## Abstract

Perception, decisions, and sensations are all encoded into trains of action potentials in the brain. The relation between stimulus strength and all-or-nothing spiking of neurons is widely believed to be the basis of this coding. This initiated the development of spiking neuron models; one of today's most powerful conceptual tool for the analysis and emulation of neural dynamics. The success of electronic circuit models and their physical realization within silicon field-effect transistor circuits lead to elegant technical approaches. Recently, the spectrum of electronic devices for neural computing has been extended by memristive devices, mainly used to emulate static synaptic functionality. Their capabilities for emulations of neural activity were recently demonstrated using a memristive neuristor circuit, while a memristive neuron circuit has so far been elusive. Here, a spiking neuron model is experimentally realized in a compact circuit comprising memristive and memcapacitive devices based on the strongly correlated electron material vanadium dioxide (VO_2_) and on the chemical electromigration cell Ag/TiO_2−*x*_/Al. The circuit can emulate dynamical spiking patterns in response to an external stimulus including adaptation, which is at the heart of firing rate coding as first observed by E.D. Adrian in 1926.

## Introduction

**Figure 1:**
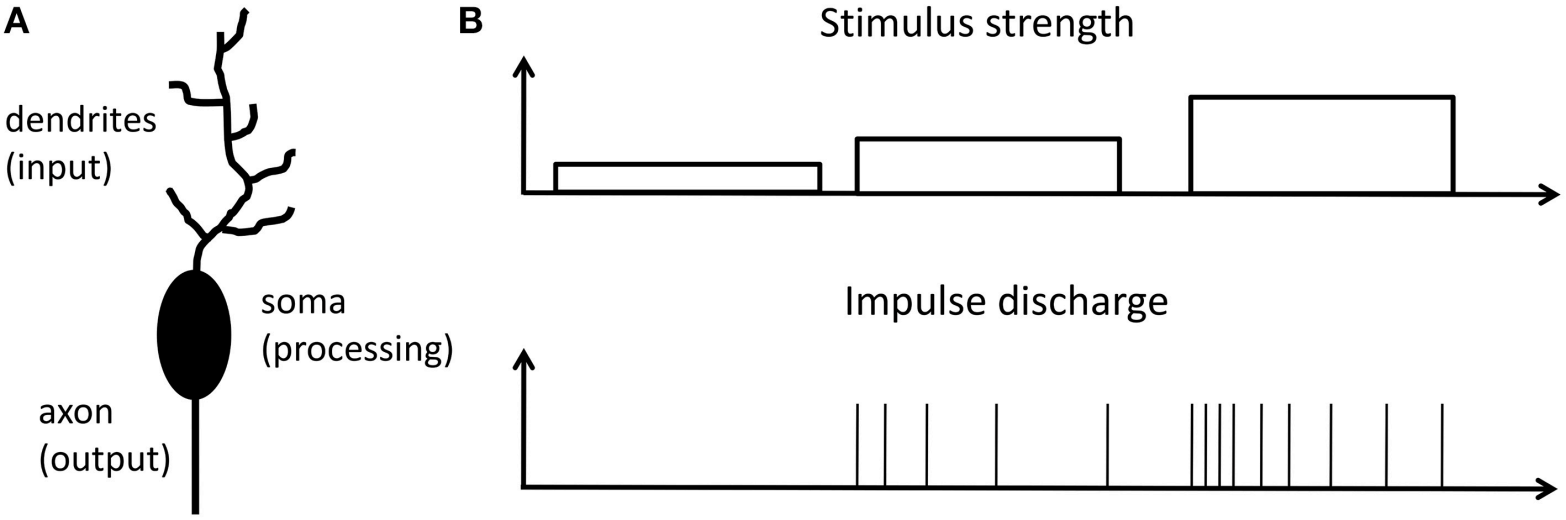
**Response to a stimulation principle: (A) Schematic of a single neuron, which can be divided into three functional parts: Dendrites, collect signals from other neurons; cell body (soma), the central processing unit of a neuron; axon, neuronal output stage**. **(B)** Relationship between firing rate of a neuron and the strength of input stimulation reflecting the response to a stimulation principle as proposed by E. D. Adrian in 1926 (Adrian, 1926, 1928; Maass and Bishop, 2001).

In a brain the most prominent processing units are neurons. An archetypical neuron consists of dendrites (the input), soma (the processing unit), and axon (the output) as schematically shown in Figure 1A. Information between neurons is encoded into sequences of identical spikes or action potentials, which appear in spatial and irregular temporal patterns. It is widely believed that in neurons input stimuli are transacted into a firing rate of action potentials at the output. This so-called firing rate hypothesis was first recognized in 1926 by E.D. Adrian (Adrian, 1926, 1928) from the investigation of sensory neurons. Nowadays the firing rate hypothesis is generalized and considered as the basic encoding scheme of neurons in the primary visual cortex, somatosensory cortex, auditory cortex, place cells in the hippocampus and many other brain regions (Barlow, 1961; Laughlin, 1989; Maass and Bishop, 2001; Gerstner and Kistler, 2002; Natelson, 2013). An additional important (and already observed by E. D. Adrian in 1926) aspect of neural signal processing is that neurons only transiently sustain a (high) firing rate, even when the stimulus is permanently applied (Adrian, 1926, 1928). In other words, the transiently decaying signal is encoded via a variable action potential firing rate, as sketched in Figure 1B. Adrian interpreted his findings as a general concept of adaptation in all living species. The decreasing firing rate, while applying a timely constant stimulus, results in reduced sensation, or in other words the species adapts to the outer world (Mausfeld, 2013). In this way sensory adaptation enables the subtraction of spatial and temporal steady signals parts, which leads to a signal renormalization and facilitates the consecutive coding. We would like to emphasize that the firing rate code hypothesis is nowadays extended even to neurons far from the receptor neuron and goes beyond the sensory transduction concept. The concept of spiking neurons is applied to neurons in mammalian brains, since those neurons are directly involved in information processing (Bear et al., 2006; Shepherd and Grillner, 2010).

In more detail, spikes or action potentials are result from voltage controlled ionic currents in cell membranes and are short electrical pulses in the millisecond range with a peak-to-peak amplitude of about 100 mV. Hodgkin and Huxley described the spike generation for the first time, by using an electronic equivalent circuit model (Hodgkin and Huxley, 1952), which is essentially based on sodium and potassium ion channels and enables the description of electrophysiological properties relevant for the spike's line shape. However, information in neural networks is encoded by the number and timing of those spikes rather than by the spike shape. Hence, simple phenomenological spiking neuron models are of interest rather than detailed conductance-based neuron models (Gerstner and Kistler, 2002), because these kind of models already enable studying neuronal coding, memory, and network dynamics in a simple circuit model (Fitzhugh, 1955; Hindmarsh and Rose, 1984; Mead, 1989; Izhikevich, 2003).

Archetypical neurons may formally be divided into three functional parts, as sketched in Figure 1A: While dendrites serve as input stage of the neuron, collecting signals from other neurons, the cell body (soma) is the central processing unit of a neuron, which generates a spike whenever the membrane potential exceeds a certain threshold *V*_θ_ defined at the axon-hillock. Finally, the axon serves as an active transmission line for the generated spikes toward post-synaptic neurons (Bear et al., 2006). A straightforward description of a neuron is given by the integrate-and-fire model (Gerstner and Kistler, 2002), where a resistor *R* is connected in parallel with a capacitor *C* and they are driven by a current *I*(t), which can be expressed as

(1) $$\begin{array}{l} \tau_{m}\frac{du\left(t\right)}{dt}=-u\left(t\right)+R\left(I,t\right)I\left(t\right), \end{array}$$

where τ_*m*_ = *RC* is the time constant of the circuit and *u(t)* refers to the membrane potential. Moreover, a threshold electronic circuit is used, so that the neuron is firing whenever *u(t)* reaches a defined threshold voltage *V*_θ_, whereafter the potential *u(t)* is reset to a reset potential *u*_*r*_ < *V*_θ_ (Gerstner and Kistler, 2002).

To realize compact, real-time, and energy efficient electronic neuron circuits, the analog complementary metal-oxide-semiconductor (CMOS) technology has been successfully employed (Indiveri et al., 2011). This approach goes back to the 80's of the last century, initiated by Carver Mead (Mead, 1989), where the integration of those circuits into very large-scale integration (VLSI) technology offers the possibility to build up real-time autonomous (cognitive) systems (Chicca et al., 2014). However, to further improve such bio-inspired circuits, non-volatile electronic, and/or ionic devices are required which improve the circuit design flexibility combined with a reduced circuit complexity. Memristive devices may fulfill these requirements. Although, theoretically predicted by Chua in 1971, researchers have just realized the useful functionalities of those devices for neural computing in the last couple of years. The ongoing research on memristive devices for neural systems mainly concentrates on the emulation of biological synapses and important synaptic functionalities (Hasegawa et al., 2010; Jo et al., 2010; Ohno et al., 2011; Zamarreño-Ramos et al., 2011; Jeong et al., 2013; Ziegler et al., 2015). In contrast, the entire neural functionality has been studied less (Pickett et al., 2013; Lim et al., 2015).

Mathematically, Equation (1) belongs to the class of *van der Pol* oscillators (van der Pol, 1926) if *R* is replaced by voltage-controlled or current-controlled devices with negative differential resistance (NDR). Recently, Pickett et al. (2013) showed that metal to insulator (MIT) phase transition materials are highly attractive for such circuits, since they allow to design inductor free circuits with the advantage of a reduced power consumption, scalability to the nanoscale, and integrability in complex neural network circuits (Lim et al., 2015). In particular, their spiking circuit is an experimental implementation of the neuristor (Crane, 1960) proposed by Hewitt Crane in 1960 and it allows emulation of some important neuronal functions, as for example the all-or-nothing spiking (Pickett et al., 2013; Lim et al., 2015). However, a neuristor cannot be considered as an electronic substitute of a neuron because a neuristor can only enable a subset of neuronal functions. In particular a neuronal coding scheme is missing, which represents the link between stimulus and response (Adrian, 1926; Chapleau, 2007), as sketched in Figure 1A.

In this work we combine the opportunities of memristive devices with phenomenological neuron circuit models to implement an analog memristive spiking neuron circuit. The circuit consists of: (1) a non-linear resistor exhibiting a NDR, (2) a memcapacitance sub-circuit derived from parallel/serial connected capacitors and a memristive device, as well as (3) a passive diode based output stage. We show that this circuit allows to realize a spiking neuronal coding scheme including firing frequency adaptation, where the amount and frequency of generated spikes are depend on the intensity and duration of an external current pulse, as well as on the number of generated spikes. Therefore, the circuit can be considered as a neuromorphic engineered version of the biological activity pattern (significance of a response) to an external stimulus principle, as first observed by A.D. Adrian in 1926 (Adrian, 1926, 1928). For the experimental realization of the neuron circuit the strongly correlated electron material VO_2_ patterned in a lateral device structure is used. We show that this device exhibits a *S*-type shape NDR in a section of his *I*-*V* curve. Moreover, a memristive behavior of the neuron circuit is obtained using a memcapacitance, which has been experimentally realized using a capacitive divider with a memristive device in parallel to the second capacitor. Here, we used Ag-doped TiO_2−*x*_ as memristive device, which inherent stochastic nature additionally introduces stochastic noise to the neuron model.

The paper is organized as follows: In the Materials and Methods Section particular features of the applied materials are summarized and the film patterning techniques for device fabrication are explained. In the subsequent Section entitled Circuit Layout and Device Characterization the engagement of the individual circuit element to function as firing rate adapter is presented. The most prominent features of our circuit, i.e., fire rate coding, adaptation, and refractoriness, are summarized in the Section Results and Discussion followed by a Conclusion.

## Materials and methods

### Device fabrication

Memristive devices used for the memcapacitive circuit were fabricated from Ag/TiO_2−x_ /Al planar capacitor structures (a sketch of the material stack is shown in the inset of **Figure 5A**). The 45 nm Ag bottom electrode was deposited by thermal evaporation on thermally oxidized Si substrates. Standard optical lithography was used to define 50 × 50 μm windows. Afterwards, an 17.6 nm thick TiO_2−x_ layer was deposited by reactive sputtering, followed by the deposition of a 140 nm Al top electrode and a subsequent lift-off in acetone.

Vanadium dioxide (VO_2_) devices were fabricated in a lateral Au/VO_2_/Au geometry, as shown in **Figure 3B**. Therefore, VO_2_ films were grown directly on single crystal TiO_2_ substrates by Pulsed Laser Deposition (PLD) using a KrF excimer laser of 248 nm in wavelength (Kim and Kwoka, 1994; Petraru et al., 2014). A commercially available sintered ceramic V_2_O_5_ target was used. During deposition, the temperature of the substrate was kept at 380°C with an oxygen pressure of 1 × 10^−2^ mbar in the PLD chamber. The energy density of the laser at the target was about 2.5 J/cm^2^. After VO_2_ thin film deposition, electrodes with a separation of 2–4 μm are defined by optical lithography, followed by the deposition of a 40 nm thick Au film and a subsequent lift-off in acetone.

### Electrical measurements

The neuron circuit was realized on a breadboard using commercially available metal-film resistors and polyester film capacitors with a tolerance of, respectively, 1 and 10%. The custom-made electronic devices are externally connected to the circuit board. Therefore, a Süss wafer prober was employed, where the individual memristive cells are electrically contacted through tungsten probe tips. The transient behavior of the neuron circuit was measured using a Tektronix TDS 7104 oscilloscope. As an input signal of the neuron circuit constant current pulses were applied. Therefore, an Agilent E5263A source measurement unit (SMU) was employed. The oscilloscope was used to record the voltage response of the circuit.

Current–voltage measurements (*I–V* curves) on single devices were obtained using an Agilent E5263A SMU by sweeping the applied current (voltage) and measuring the voltage (current) simultaneously. Additionally, a current (voltage) compliance was set in order to avoid a breakdown of the investigated device by electrical stress.

## Circuit layouts and device characterizations

### Memristive spiking neuron circuit

**Figure 2:**
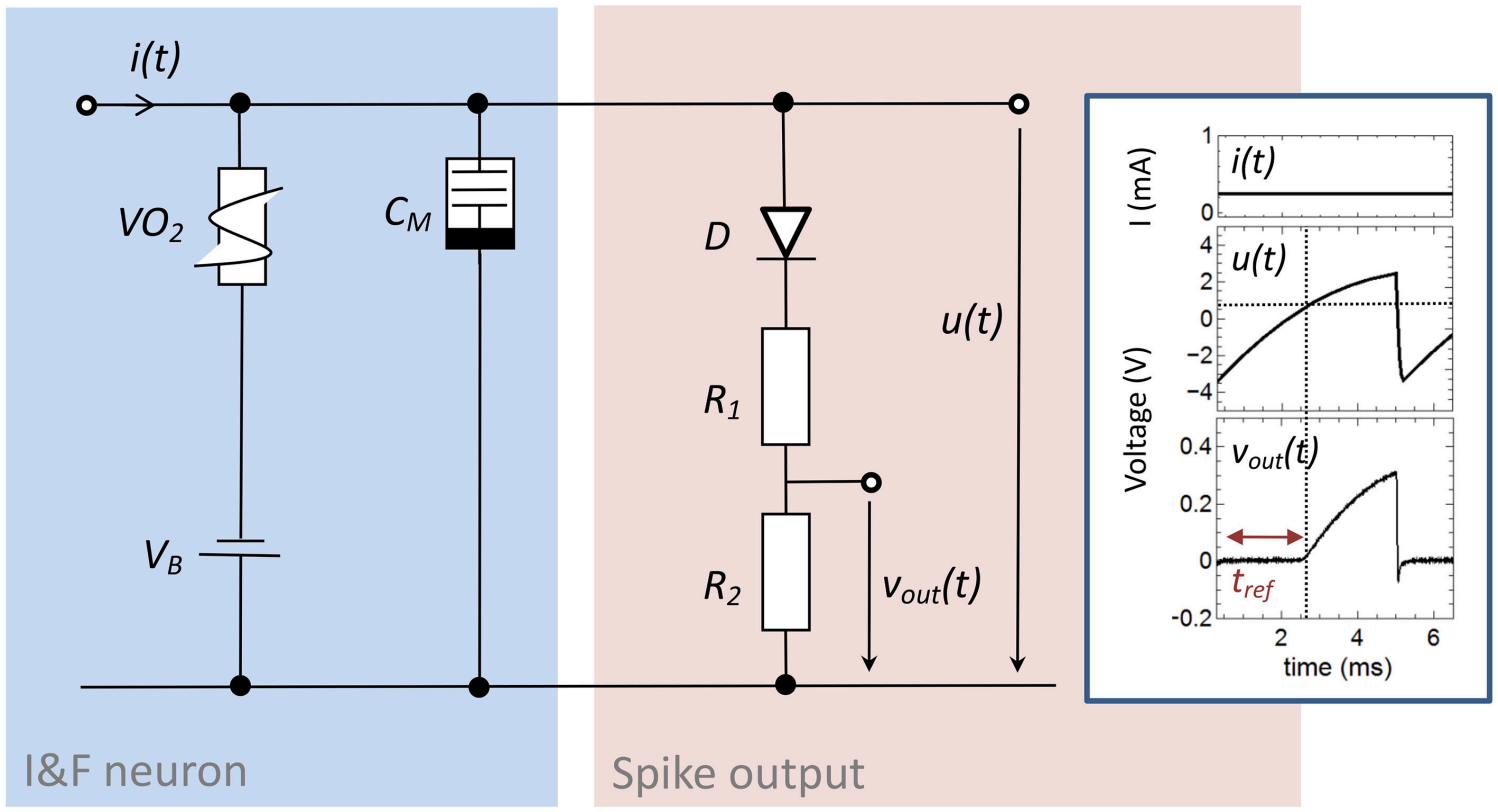
**Circuit scheme used to emulate neuronal functionalities: The circuit consists of an integrator circuit (blue) based on a negative differential resistor and a memcapacitor ***C***_***M***_, as well as on an output branch with a diode ***D*** in series with two ohmic resistors ***R***_1_ and ***R***_2_ (red) which deliver the spikes ***v***_***out***_***(t)*** from the oscillating voltage ***u(t)*****. Inset: Typical oscillation for a constant input current of 0.25 mA. While the constant voltage source *V*_*B*_ shifts the base voltage to a negative value, the voltage divider with the diode *D* cause a constant output voltage *v*_*out*_*(t)* in an interval (labeld as *t*_*ref*_) in which *u(t)* is smaller than the build-in voltage of the diode. The used device parameters of the circuit were *R*_1_ = 47 kΩ, *R*_2_ = 10 kΩ, *C*_*M*_ = C_0_ = 0.068 μF, *V*_*B*_ = −3.5 V.

The here proposed memristive spiking neuron circuit is shown in Figure 2. The main parts of this circuit are an integrator circuit (in accordance to Equation 1) with a negative differential resistor (blue box in Figure 2) and a spike output branch (red box in Figure 2) used to generate the output spike *v*_*out*_*(t)* from the oscillating voltage *u(t).* In particular, the negative differential resistor causes a breakdown of *u(t)*, when its negative differential regime is reached. This results in an oscillation of *u(t)* during constant current input *i(t)*. Furthermore, a memcapacitance *C*_*M*_ is used for the integrator circuit, which allows varying the frequency of oscillation in dependence of the charge flow history, i.e., the number of generated voltage spikes. Thus, *C*_*M*_ defines the memristive behavior of the proposed neuron circuit model. At the output stage of the neuron circuit the serial connection of *D* with *R*_1_ and *R*_2_ allow to emulate a desired line shape of the generated output voltage spikes *v*_*out*_*(t)* across *R*_2_ (cf. Figure 2), including a refractory period (labeled by *t*_*ref*_ in the inset of Figure 2). A constant voltage source *V*_*B*_ is added in series to the negative differential resistor which allows resetting the capacitance of *C*_*M*_ after the external current stimulus disappears. The inset of Figure 2 shows typical obtained voltage characteristics for *v*_*out*_*(t)* and *u(t)* for a constant current input *i(t)* = *i*_0_. In detail, the voltage source *V*_*B*_ causes a shift of *u(t)* to a negative base voltage, while the diode *D* builds with *R*_1_ and *R*_2_ a voltage divider which helds *v*_*out*_*(t)* constant (defining *t*_*ref*_) as long as *u(t)* is smaller than the build-in voltage of *D* (*V*_*D*_ = 0.7V). If *u(t)*, however, exceeds *V*_*D*_ a spike *v*_*out*_*(t)* is initiated with a spike width of ~2 ms, which is in accordance to biological spike times.

The key devices of this circuit are the strongly correlated electron material vanadium dioxide (VO_2_), which provides a NDR and the memcapacitor *C*_*M*_, which has been realized by a sub-circuit containing a capacitive divider and an Ag/TiO_2−x_/Al electrochemical metallization cell (cf. **Figure 4A**). Both devices are discussed in the following first, before going deeper in the analysis of the neuron circuit. In the following both devices are discussed individually, with a subsequent deeper analysis of the complete neuron circuit in the Results and Discussion section.

### VO_2_-based negative differential resistor

**Figure 3:**
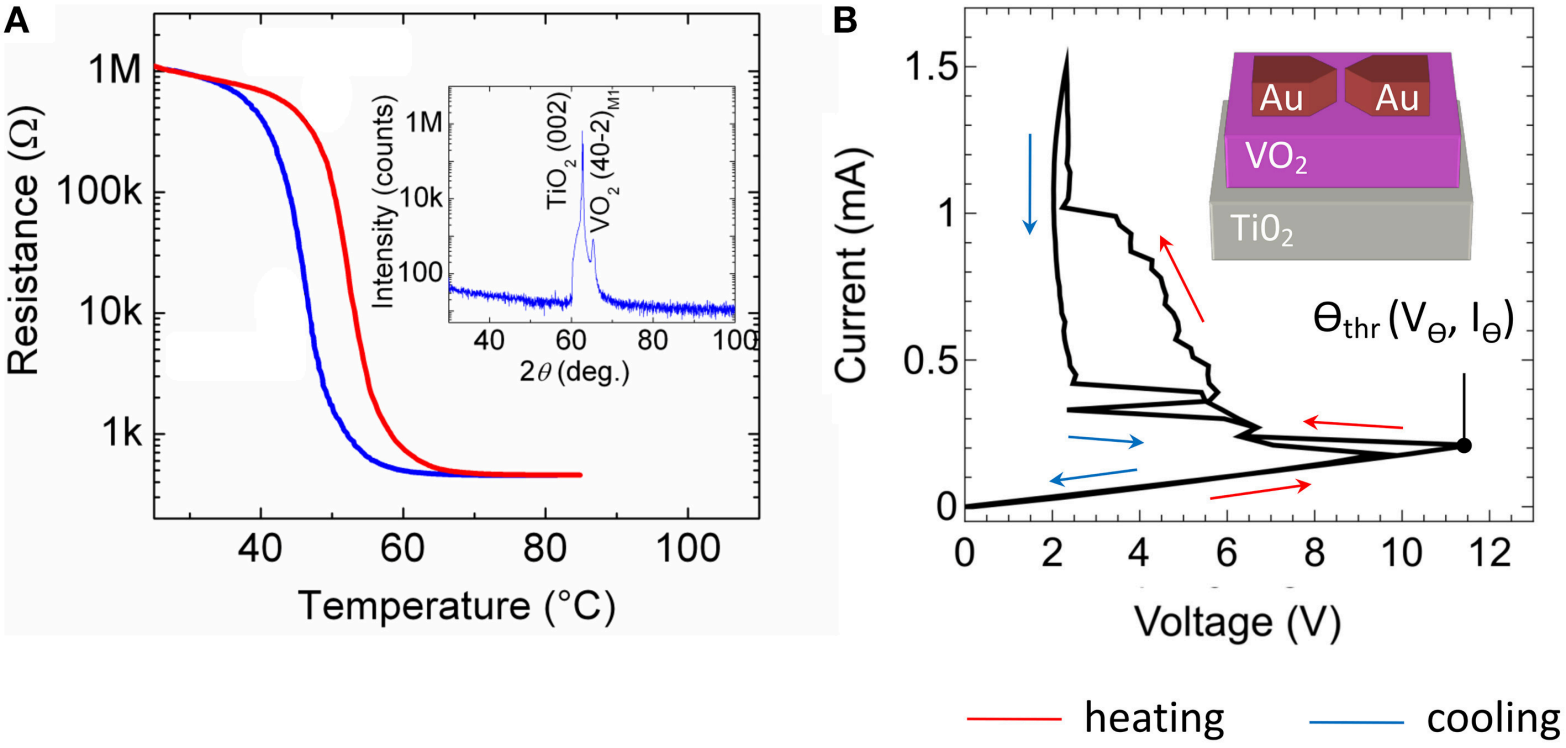
**Electrical characteristics of the VO_2_ device: (A) Resistance versus temperature characteristic**. Inset of **(A)**: X-ray *2*θ*-*ω scan of the VO_2_ film on a TiO_2_ (001) substrate measured with Cu K_α_ radiation. The observed peaks correspond to TiO_2_ (002) and to VO_2_ (40-2)_M1_ (insulating phase, space group P2_1_/c, *a*_*M*1_ = 5.743 Å, *b*_*M*1_ = 4.517 Å, *c*_*M*1_ = 5.375 Å, and β_*M*1_ = 122.618°) **(B)** Current voltage characteristic of a lateral VO_2_ device together with a schematic sketch of the device structure. In all sub-figures indicating the red arrows the heating cycle and the blue arrows showing the cooling direction.

As resistor *R* for the presented spiking neuron circuit (cf. Figure 2) a current-controlled VO_2_ device was used, which exhibits a NDR. In particular, VO_2_ belongs to the class of strongly correlated electron materials and exhibits a MIT phase transition (Mott—Peierls transition) at approximately 60°C (Morin, 1959; Nakano et al., 2012; Natelson, 2013) accompanied by a structural phase transition from the high-temperature tetragonal phase to the low-temperature monoclinic phase. A typical resistance vs. temperature curve of the here fabricated VO_2_ film is shown in Figure 3A. A constant voltage of 1 V was applied to the film, while the current was recorded simultaneously. The temperature was ramped from 30 to 95°C and back to 30°C. In particular, we found the Mott transition temperature at 58°C during heating (red curve in Figure 3A) together with a hysteresis of ~10°C during cooling (blue curve in Figure 3A). Moreover, a resistance change of more than four orders in magnitude was observed which reflects a good quality of the VO_2_ film. The quality of the VO_2_ could be further verified by using *X*-ray diffraction spectroscopy (see inset of Figure 3A), which exhibits peaks corresponding to the (001) crystal orientation of the TiO_2_ substrate and to the VO_2_ (40-2)_M1_ monoclinic phase M1, as expected at room temperature (Andersson, 1956).

Instead of increasing the substrate temperature to introduce the Mott transition also electrical stress can be used, where Joule heating of local filamentary grain structures has been identified as the origin of the resistance switch (Driscoll et al., 2012; Guénon et al., 2013). Recently, such two terminal devices, in which the Mott transition can be introduced by electrical stress, were identified as memristive devices (Chua, 1971) with a transient memory (Pickett et al., 2013). A typical current-voltage characteristic of our device is depicted in Figure 3B together with a sketch of the device structure. Therefore, the applied current was ramped between 0 and 1.5 mA, while simultaneously the voltage was measured. Starting from the initial high resistance of the device (${\text{R}}_{\text{VO}2}^{\text{H}}$ = 55 kΩ), the device resistance decreases gradually to a lower resistance of ${\text{R}}_{\text{VO}2}^{\text{L}}$ = 2–14 kΩ if the applied current-voltage threshold Θ_thr_ is exceeded (cf. Figure 3B).

### Ag-doped TiO2_-x_ -based memcapacitive device circuit

**Figure 4:**
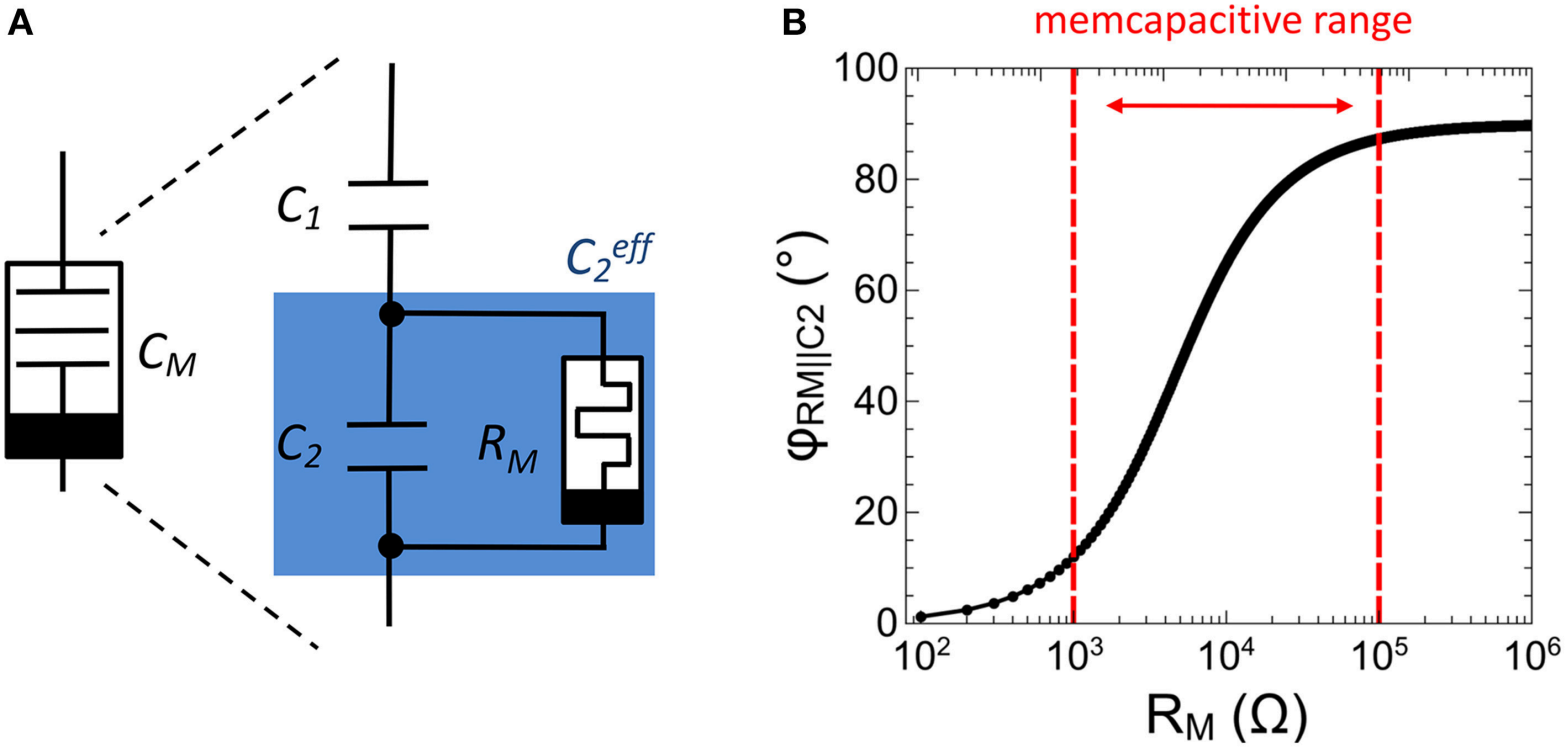
**Experimental realization of a memcapacitor: (A) Schematic drawing of the memcapactitive circuit including a memristive device**. **(B)** Calculated impedance phase φ_*RM*||*C*2_ between the memristive device and the capacitor *C*_2_ as function of the resistance *R*_*M*_.

In analogy to memristive devices (Chua, 1971), memcapacitors are defined by a capacitance which depends on the charge flow history, i.e., *C*_*M*_ = *C*_*M*_*(q,t)*. The memcapacitor was theoretically proposed by Di Ventra et al. (2009) in 2009 and shows promising functionalities for adaptive circuits (Traversa et al., 2013). To experimentally realize a memcapacitance, we used the sub-circuit shown in Figure 4A. This circuit consists of two individual capacitances *C*_1_ and *C*_2_ arranged in a capacitive divider with a single memristive device *R*_*M*_ connected in parallel to *C*_2_, (labeled as $C_{2}^{eff}$ in Figure 4). In this configuration the resistance of the memristive device can be varied in accordance to the charge flow history, i.e., *R*_*M*_ = *R*_*M*_*(q,t)*. The therefore obtained memcapacitive behavior (depending on the resistances of *R*_*M*_) can be seen by regarding the total capacitance *C*_*M*_ of the sub-circuit, which can be expressed as

(2) $$\begin{array}{l} C_{M}=\frac{C_{1}C_{2}^{eff}}{C_{1}+C_{2}^{eff}}\text{ with }C_{2}^{eff}\left({u_{m},R}_{M},t\right)\\ =\frac{1}{u_{m}}\left(q\left(t\right)-q_{m}\left({u_{m},R}_{M},t\right)\right). \end{array}$$

Here *u*_*m*_ defines the voltage drop across $C_{2}^{eff}$, while *q* and *q*_*m*_ are the total charge of the capacitive branch and the charge stored in between *C*_1_ and $C_{2}^{eff}$, respectively. Hence, $C_{2}^{eff}$ depends on *R*_*M*_*(q,t)* which provides a memcapactive behavior as proposed theoretically by Di Ventra et al. (2009).

In order to gain some more insight into the functionality of the memcapacitive circuit and to give advices for the design of memristive devices it is worth to look at the total impedance of $C_{2}^{eff}$, which reads.

(3) $$\begin{array}{l} Z_{R_{M}||C_{2}}\left(R_{M}\right)=\frac{1}{\frac{1}{R_{M}}\;+j\omega C_{2}}. \end{array}$$

In particular, Equation (3) implies that $C_{2}^{eff}$ (and therewith *C*_*M*_) is affected whenever the impedance phase φ_*RM*||*C*2_ between *C*_2_ and *R*_*M*_ is less than 90°. However, to fulfill this condition *R*_*M*_ must be varied in accordance to *C*_2_. In this investigation *C*_1_ and *C*_2_ have been selected in respect to biological time scales, which is for a single spike in the range of a few *ms*. By further taking the resistance of the VO_2_ negative differential resistor (cf. Figure 3) into account, *C*_1_ and *C*_2_ have been chosen to 0.165 and 0.068 μF, respectively. Hence, to ensure that Δφ_*RM*||*C*2_ is less than 90°, *R*_*M*_ must be variable in between 1 and 100 kΩ, as depicted in Figure 4B. In particular, the impedance phase of $C_{2}^{eff}$ will be most sensitive to *R*_*M*_ changes when *R*_*M*_ and *C*_2_ contribute to the overall impedance magnitude roughly equally. As we will show as next, a Ag-doped TiO_2−x_ based memristive device fulfills this requirement.

**Figure 5:**
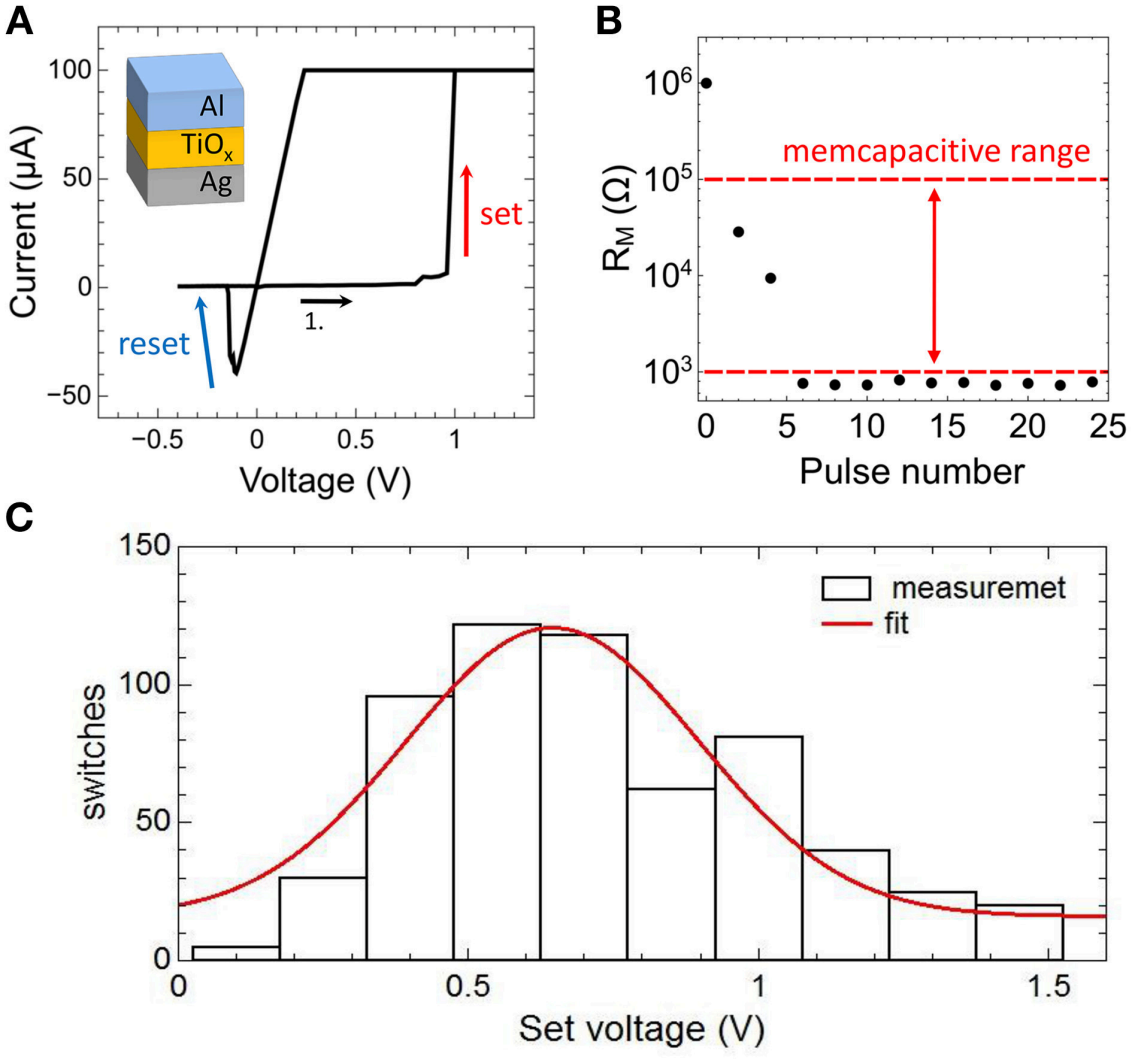
**Electrical characteristics of the memristive device: (A) Measured ***I-V*** curve of an Ag-doped TiO_2-x_ -based memristive device together with a sketch of the layer sequence of this cell**. A current compliance of 0.1 mA has been set. **(B)** Measured resistance variation of the device by applying 2 ms voltage pulses with an amplitude of 10 V. The dashed red lines show the desired memcapacitive range obtained from Figure 4B. **(C)** Distribution of the set voltage obtained from 620 identically voltage sweeps using a current compliance of 0.1 mA. The red curve is a Gaussian data fit.

In Figure 5A typical current-voltage characteristic (*I*-*V* curve) obtained on a single Ag/TiO_2−x_/Al memristive device is shown together with a sketch of the device structure. By sweeping the bias voltage between 1.4 and –0.5 V the device resistance changes at a positive set voltage of *V*_*set*_ = 0.95 V from the initial high resistance state of 1 MΩ to the low resistance state of 1 kΩ and vice versa at negative voltage at a reset voltage of *V*_*Reset*_ = −0.2 V. In order to avoid a device breakdown a current compliance of 0.1 mA was set. To analyze the resistance switching of the memristive device in some more detail single voltage pulses of 10 V in height and 2 ms in width are applied to an individual Ag/TiO_2−x_/Al cell. In particular, that voltage pulse corresponds to the maximal possible voltage which can be dropped across the memristive cell when the cell is operating in the neuron circuit of Figure 2. The obtained change in resistance is shown in Figure 5B. We found that the device resistance is decreased from initially 1–0.8 kΩ under such voltage pulses. Therefore, the recorded resistances are within the memcapacitive interval estimated from Equation (3) and marked by two red dashed lines in Figures 4B, 5B.

We would like to mention that the resistive switching process in the used Ag/TiO_2−x_/Al memristive cells has an inherent stochastic nature (Gaba et al., 2013), where the needed number of voltage pulses to set the device resistance depends on local Ag migration processes. A more detailed analysis of the stochastic behavior of the used Ag/TiO_2−x_/Al memristive device is shown in Figure 5C. Therein, the set voltages *V*_*set*_ of 620 consecutive current-voltage cycles are shown, which has been obtained from *I*-*V* measurements in which the voltage was ramped from 0 to 2 V and a current compliance of 0.1 mA was set. By fitting the experimental data with a Gaussian function, an average set voltage of *0.64 V* was obtained with a full width at half maximum of *0.25 V*. In particular, the device stochastic influences the transient dynamics of the neuron circuit, as we will discuss it below.

## Results and discussion

In the following we discuss the memristive spiking neuron model (depicted in Figure 2) in detail. For a clearer presentation of this circuit we split the analysis in two parts. First, we discuss the emulation of fire-rate coding, i.e., how the circuit emulates dynamical spiking patterns in response to an external stimulus. Thereafter, the emulation of adaptation and refractoriness will be addressed. We therefore like in particular to focus on the adaptive/memristive behavior of our neuron circuit.

### Firing rate coding

**Figure 6:**
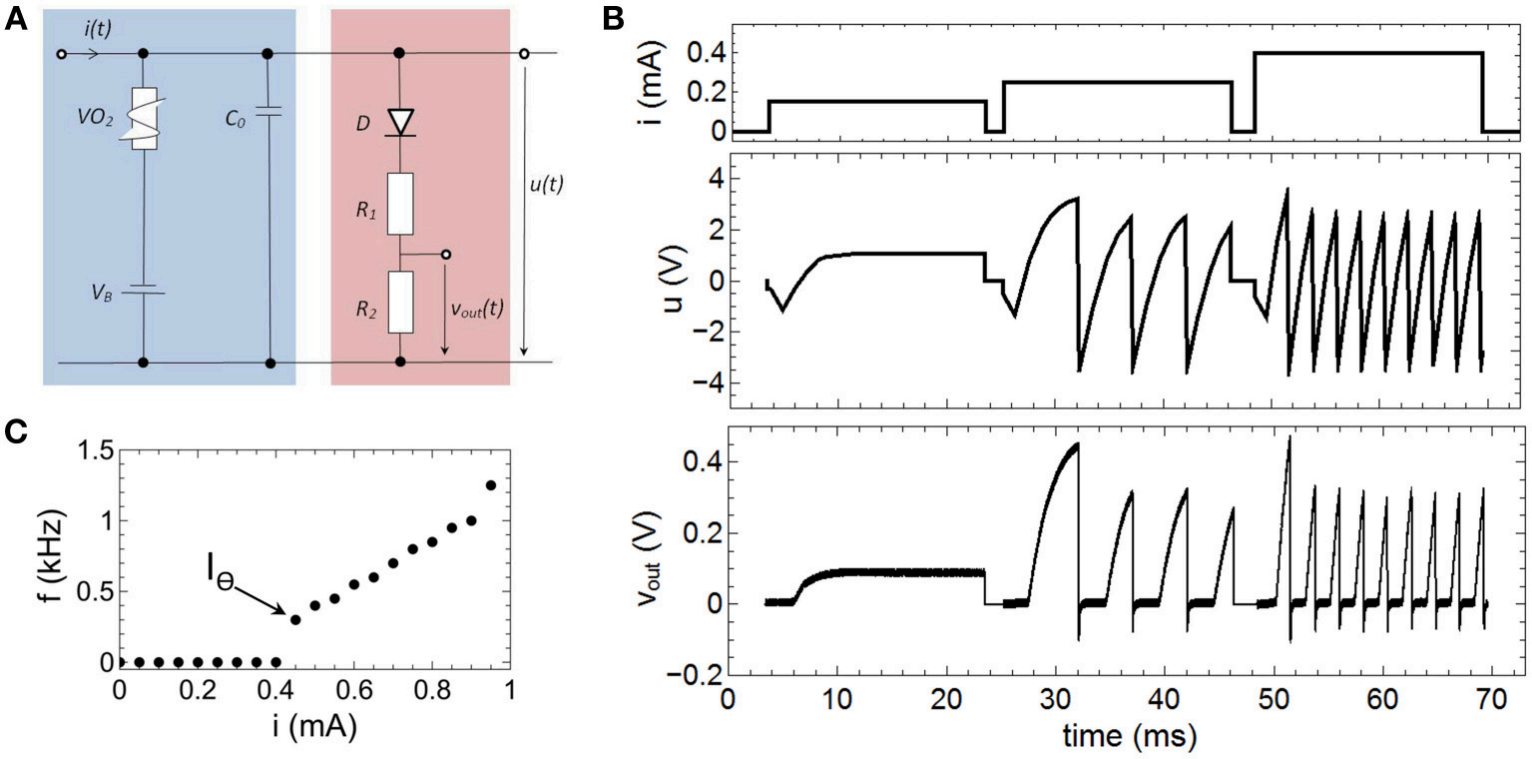
**Emulation of fire frequency coding: (A) Circuit layout to emulate firing frequency coding**. In contrast to Figure 2 *C*_*M*_ was replaced by a constant capacitance *C*_**0**_. **(B)** Recorded spike pattern for different current inputs. **(C)** Measured oscillation frequencies as function of the input current *i(t)*. *I*_Θ_ denotes the threshold value for the spike initiation. Parameters used for the circuit: *R*_1_ = 47 kΩ, *R*_2_ = 10 kΩ, *C*_0_ = 0.068 μF, *V*_*B*_ = −3.5 V.

The relevant mechanisms for the emulation of spike rate coding of our proposed neuron circuit scheme (Figure 2), can be dissected in that the memcapacitance (*C*_*M*_) is fixed. By replacing the TiO_2_ based sub-circuit with a constant capacitor *C*_0_ this can be achieved and we obtain a circuit as shown in Figure 6A. The therewith recorded voltage characteristics *u(t)* and *v*_*out*_*(t)* for different current inputs *i(t)* are shown in Figure 6B, while the used parameters of the circuit devices read as *R*_1_ = 47 kΩ, *R*_2_ = 10 kΩ, *C*_*M*_ = *C*_0_ = 0.068 μF, *V*_*B*_ = −3.5 V. As a result we found that a current strength up to 0.15 mA will affect no spike generation within the investigated time interval, while current strengths of 0.25 and 0.4 mA triggers the circuit to spike with different numbers of spikes. In order to study this point in some more detail, single constant current pulses of 20 ms and amplitudes ranging from *0* to 0.95 mA were applied to the neuron circuit. The circuit produced fire frequencies (number of spikes per second) as function of the applied currents are depicted in Figure 6C. While for *i(t)* smaller then the VO_2_ threshold current *I*_Θ_ (cf. Figure 3B) no oscillations are evoked, input currents above *I*_Θ_ generate a spiking of the circuit. Moreover, the generated frequency of spikes increased linearly with increasing current which allows to directly relat the spike frequency to the intensity of the stimulus. This represents the biological firing rate mechanism, as sketched in Figure 1B (Adrian, 1926; Chapleau, 2007). In technical terms, the linear increase in the fire frequency is related to the slope of the negative differential branch of the *VO*_2_ device (cf. Figure 3B). Consequently, the total interval for the frequency coding is defined by the length of the negative differential branch which is for the here realized VO_2_ cells for current strengths varying in between 0.4 and 0.95 mA. Because the firing rate is constant for a constant given stimulus (i.e., the firing rate exhibits no transient decay), the onset *f*_0_(I) and steady *f*_∞_(I) curve are identical (Benda and Herz, 2003).

### Adaptation and refractoriness

As furthermore recognized in 1926 by Adrian (Adrian, 1926, 1928), the firing rate of neurons transiently decreases rather than staying constant (cf. Figure 1B), if both the receptor and organisms habituate to a persistent stimulus. In particular, Adrian expected that the post transient decrease in the firing rate might reflect the degree of habituation of the stimulus, i.e., adaptation of an external stimulus (Adrian, 1928). Nowadays, adaptation is believed to be the essential process of a signaling system to be better suited to environmental changes and it can be observed at nearly any level of biological systems (Maass and Bishop, 2001). In the spike trains of regularly firing neurons, the adaptation of their firing frequency during sustained current input is believed to be fundamental in forward masking, selective attention, and in the synchronization of neuronal assemblies (Maass and Bishop, 2001; Fuhrmann et al., 2002).

**Figure 7:**
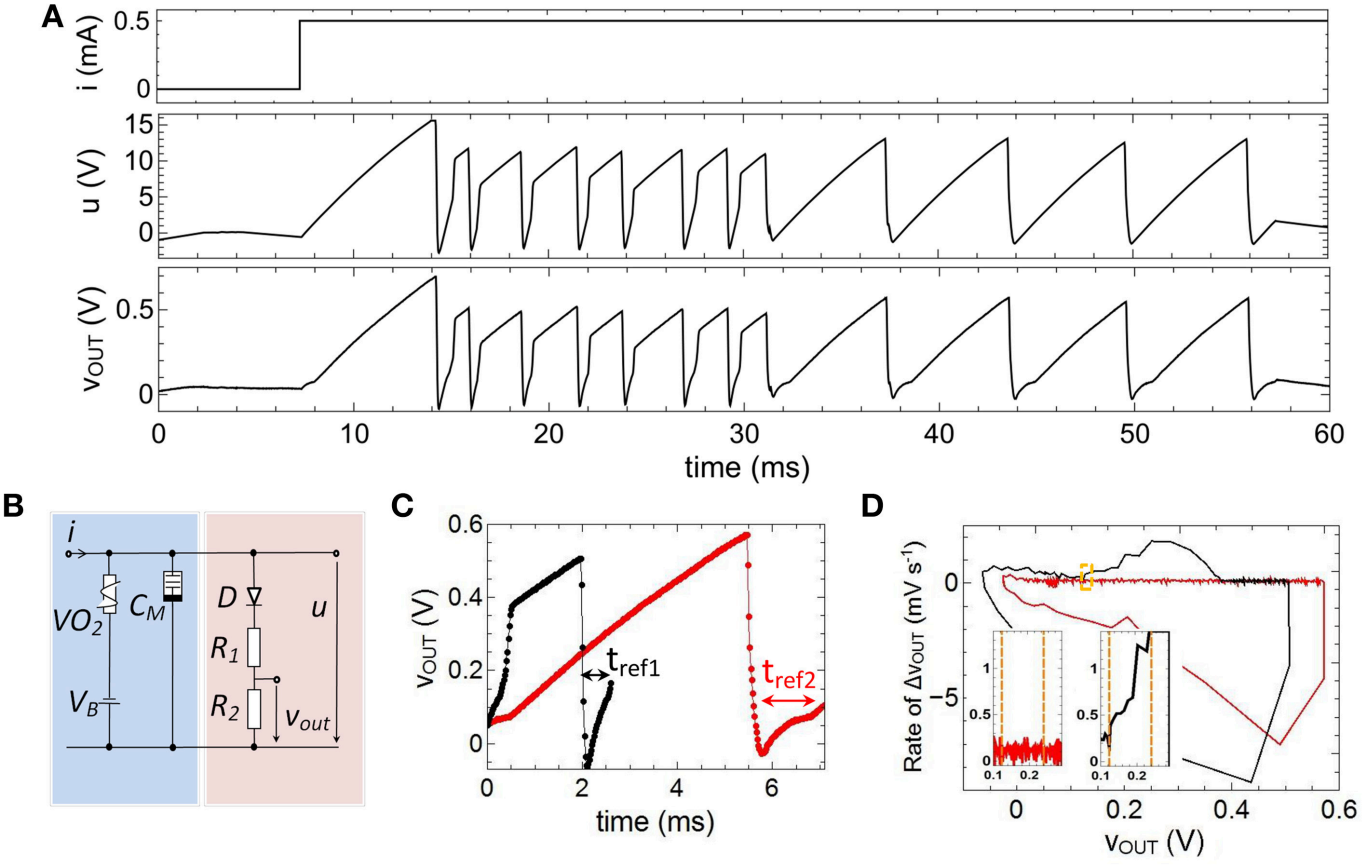
**Emulation of adaptation: (A) Measured spike pattern for a constant current input based on a memcapacitance ***C***_***M***_***(t)*****. **(B)** Layout of the investigated circuit which corresponds to the one depicted in Figure 2. **(C)** Characteristics of two individual spikes and corresponding phase diagram **(D)**, where the insets show the initial phase of the spikes. Parameters used for the circuit: *R*_1_ = 1*MΩ*, *R*_2_ = 47*kΩ*, *C*_1_ = 0.165μF, *C*_2_ = 0.068μF, *V*_*B*_ = −5.5 V.

In order to emulate fire frequency adaptation the capacitance *C*_0_, shown in Figure 6A, has been replaced by a memcapacitance *C*_*M*_ = *C*_*M*_*(t)* (cf. Figure 4) which leads to the circuit presented in Figure 2 and Figure 7B. In Figure 7A the therewith obtained voltage characteristics for *u(t)* and *v*_*out*_*(t)* for a constant current input of *0.5 mA* are presented. The used device parameters of the circuit were *R*_1_ = 1 MΩ, *R*_2_ = 47 kΩ, *C*_1_ = 0.165 μF, *C*_2_ = 0.068 μF, *V*_*B*_ = −5.5 V. As a main result we found that the frequency of spike initiation is clearly decreased after the first eight spikes, while the amplitude of the individual spikes is nearly unaffected. This finding can also be observed in Figure 7C, where a close-up view of two of the spikes of Figure 7A is shown. While the black curve in Figure 7C corresponds to one of the first spikes of the voltage course of *v*_*ou*__*t*_ (compare also Figure 7A), the second spike (red curve) is cut from the last part of Figure 7A. In particular, their amplitudes of roughly 0.36 V and their resting potentials of *u*_*r*_ = −*15 mV* are varying slightly, while their particular spike widths and refractory period widths vary significantly. This differences in the spike width can be directly related to Equation (1), where the memconductance (cf. Equation 2) introduces a memristive time constant τ_*m*_ = *R*_*VO*2_*(i,t) C*_*M*_*(R*_*M*_*,u*_*m*_*,t)* for the current integration. The effect of such a memristive time constant can be further analyzed from experimental data (depicted in Figure 7C) by regarding the corresponding phase plots, as shown in Figure 7D. From this plot it can be seen that the rate of the voltage change of *v*_*out*_ varies little. However, the initial phases vary significantly when *C*_*M*_ changes, as it can be seen from the enlarged parts of the phase plots, depicted in the insets of Figure 7D (see yellow frame). In particular, we observed a much stronger rise in the rate of the voltage change of *v*_*out*_ for the initial spike (black curve) compared to the final spike (red curve). In this context it is worth mentioning that these features are also observed in cortical neurons and it is believed that the dynamics of spike initiation is a unique feature, which can qualitatively change the nature of neuronal encoding (Naundorf et al., 2006).

We would like to remark that in contrast to biological findings an abrupt switch rather than a gradually, continuous decrease of the fire frequency was obtained. This, in fact, belongs to the switching mechanism of the memristive device and might change if the Ag/TiO_2−x_/Al cell is replaced by a memristive device which shows a more gradual change in device resistance. However, the principles of the proposed adaptation emulation can be directly transferred to other types of memristive devices. A further important aspect of the used memristive cell is the inherent stochastic nature of the resistance switching process (cf. Figure 5C). In particular, this device behavior introduces stochastic noise to both the pulse width of a single spike and the number of spikes needed before a pulse frequency adaptation occurs (cf. Figure 7A). For example, for the spike course shown in Figure 7A, the width of the second and third spike is larger than those of spike number four, while a “real” adaptation of the firing frequency was obtained after eight voltage spikes. However, such a stochastic behavior might be of interest for a variety of network applications and are in agreement with biological neurons (McDonnell and Ward, 2011).

In general, adaptation of the neurons firing rate has to be balanced with refractoriness. Refractoriness is a general characteristic of neurons to ensure that consecutive spikes are not overlapping and defines the (forward) direction of spikes in nerve cells. In order to incorporate a refractory period, a diode was connected in series with *R*_1_ and *R*_2_ in the output branch of the neuron circuit of Figure 2 (red column). Further of importance for the emulation of a refractory period was a negative base voltage which has been generated by the constant voltage source *V*_*B*_ (cf. Figure 2). In particular, *V*_*B*_ causes a negative offset of the circuit induced oscillation of *u(t)*, while the diode *D* affects that all voltages of *u(t)* smaller than the built-in voltage of the diode (*V* = 0.7 V) leading to a constant output voltage *v*_*out*_*(t)* (see for example inset of Figure 2). Therefore, the resistances *R*_1_ and *R*_2_ have been chosen much smaller than resistance value of the diode below the built-in voltage, so that most of the voltage *u(t)* is dropping across *D* below 0.7 V. A refractory period can then be defined by the time interval for which *v*_*out*_*(t)* is stabilized by the diode (labeled as *t*_*ref*1,__2_ in Figure 7C), i.e., the time at which *u(t) is* smaller than 0.7 V. Hence, in the framework of an *I*-*F* neuron model (according to Equation 1) the built-in voltage of the diode defines the threshold voltage for the spike initiation.

## Conclusion

In conclusion, a memristive spiking neuron circuit has been experimentally realized by using a VO_2_-based negative differential resistor and a memcapacitor based on an Ag/TiO_2−x_/Al memristive cell. The circuit allows emulation of basic neuronal functionalities, including spike coding, firing frequency adaptation in real time and shows a refractory period. Moreover, the obtained spike times are consistent with the spike duration in biological systems. Further, we have shown that the combination of a memristive device and a capacitive divider allows to experimentally realizing a memcapacitance. Therewith, we were able to show that the use of a memcapacitance in an NDR oscillator allows to mimic dynamic neuronal components in which the circuit induced oscillation is changing in dependence of the charge flow history, i.e., on the number of spikes generated before. Thus, the use of a memcapacitance introduces a memristive behavior of an *I*-*F* neuron. Since such spiking neuron models are important conceptual tools for the analysis and emulation of neuronal dynamics, a memristive neuron might open important new opportunities for the realization of neuronal networks.

### Conflict of interest statement

The authors declare that the research was conducted in the absence of any commercial or financial relationships that could be construed as a potential conflict of interest.
